# Expression Dependence in the Perception of Facial Identity

**DOI:** 10.1177/2041669517710663

**Published:** 2017-06-01

**Authors:** Annabelle S. Redfern, Christopher P. Benton

**Affiliations:** School of Experimental Psychology, University of Bristol, Bristol, UK

**Keywords:** visual perception, face perception, facial identity, facial expressions, expression dependence

## Abstract

We recognise familiar faces irrespective of their expression. This ability, crucial for social interactions, is a fundamental feature of face perception. We ask whether this constancy of facial identity may be compromised by changes in expression. This, in turn, addresses the issue of whether facial identity and expression are processed separately or interact. Using an identification task, participants learned the identities of two actors from naturalistic (so-called *ambient*) face images taken from movies. Training was either with neutral images or their expressive counterparts, perceived expressiveness having been determined experimentally. Expressive training responses were slower and more erroneous than neutral training responses. When tested with novel images of the actors that varied in expressiveness, neutrally trained participants gave slower and less accurate responses to images of high compared with low expressiveness. These findings clearly demonstrate that facial expressions impede the processing and learning of facial identity. Because this expression dependence is consistent with a late bifurcation model of face processing, in which changeable facial aspects and identity are coded in a common framework, it suggests that expressions are a part of facial identity representation.

Constancy of facial identity is a fundamental ability of our face processing system, enabling us to recognise familiar faces over a variety of different appearances (Bruce, 1982). This feature is paralleled in the object recognition literature by the concept of object constancy, which concerns how we are able to recognise objects across varying retinal descriptions. Maintaining constancy, whether of objects or facial identity, is computationally demanding and requires our visual system to balance conflicting demands. It must achieve the specificity necessary to recognise categories whilst simultaneously generalising when appearance varies substantially – caused by, for example, lighting, position, size and viewpoint. Facial identity constancy is additionally challenged by facial expressions.

Explaining how identity constancy is achieved has been influential in shaping models of face perception because constancy is determined by the relationship between how we process invariant and changeable aspects of faces: Is their processing separate, or separable? The polar positions in this debate are, on the one hand, dual-route theories that advocate functionally independent processing (e.g. Bruce & Young, 1986; Haxby, Hoffman, & Gobbini, 2002, 2000); and on the other hand, models proposing that the streams processing invariant and changeable face aspects bifurcate at a later stage (e.g. Calder, 2011). Late bifurcation models thereby permit that expressions can be a part of facial identity and can predict the interaction of these facial properties. They suggest that we are able to process identity and changeable aspects separately, but that interactions between the two may arise (Calder, 2011). In contrast, dual-route theories do not readily predict such interactions.

The consensus emerging from this field is of a more complex relationship between changeable aspects and identity processing than dual-route models propose (Calder & Young, 2005; Johnston & Edmonds, 2009). Evidence weighs in favour of functional asymmetry (Calder, 2011), with expressions more dependent on identity (e.g. Fox, Oruç, & Barton, 2008). This asymmetry is perhaps because, as Calder and Young (2005) suggest, expression processing relies more on integrative mechanisms – since changeable facial cues are inherently linked with perceptual dimensions such as motion and vocalisations. This is not the only view, however, and some studies suggest a more symmetric interaction (e.g. Fisher, Towler, & Eimer, 2016).

A related debate concerns the nature of the stored representation of an individual’s facial identity. One theoretical position is that our representation is akin to a central tendency, or prototype, which is developed and refined over successive viewings of that individual’s face (e.g. Burton, Jenkins, Hancock, & White, 2005). An alternative suggestion is that our representation comprises a series of stored examples of an individual’s face, and recognition is achieved when a perceived face is a close match to a stored example (e.g. Longmore, Liu, & Young, 2008). Distinguishing between these two explanations has proved challenging because they make similar predictions (Burton, Jenkins, & Schweinberger, 2011).

Evidence suggests that simultaneously processing both social and identity information should not compromise identity processing. Although a bias to attend to expressive faces has been detected (Palermo & Rhodes, 2007), the literature indicates that expressions *facilitate* identification (Gallegos & Tranel, 2005) and face discrimination learning (Lorenzino & Caudek, 2015), and do not distract judgements of facial identity (Baudouin, Martin, Tiberghien, Verlut, & Franck, 2002; Spangler, Schwarzer, Korell, & Maier-Karius, 2010). Indeed, Bruce (1994) suggests that expressions may actually facilitate identity discriminations by helping a system to distinguish relevant variations between individuals from irrelevant variations within individuals. Variation may give rise to stability by defining boundaries (Vernon, 1952) and could prove critical to how our recognition system maintains identity constancy. Therefore investigating variation, rather than controlling it, is essential if we are to understand how we recognise people (Burton, 2013). For this reason, we developed ‘ambient’ images for use in this study. These are unmanipulated photographs of real faces taken from the environment that capture a wide range of within-person variability (Burton, 2013; Jenkins, White, Van Montfort, & Burton, 2011).

We address the issue of whether facial identity and expression are processed separately or whether they interact, by asking whether our constancy of facial identity may be compromised by changes in expression. The evidence we present, of expression-dependent performance, suggests inter-related processing of identity and expression.

## Experiment 1

### Method

#### Overview of experimental design

Idiosyncratic variability is fundamental to learning facial identities (Kramer, Ritchie, & Burton, 2015), an idea supported by face learning studies (e.g. Andrews, Jenkins, Cursiter, & Burton, 2015; Dowsett, Sandford, & Burton, 2016; Murphy, Ipser, Gaigg, & Cook, 2015). We developed and used a database of ‘ambient’ face images incorporating extensive within-person variation. We specifically included images of facial expressions, varying extensively in emotional affect and intensity.

The experiment comprised training and test phases, separated by a filler task. Participants were randomly assigned to one of two training conditions: expressive or neutral. Training involved presentations of multiple images from our database of two unfamiliar identities, but conditions differed: neutral training used images rated ‘low’ in perceived expressiveness (i.e. <50%) and expressive training used images perceived as ‘high’ (>50%) in expressiveness.

The training phase provided face learning; however, Blocks 2 to 4 repeated the images used in the initial training block. Therefore, simply remembering the responses to repeat images could explain any improvement. To address this confound, the test phase used novel images of the learnt identities. Testing was the same for all participants irrespective of training condition, thereby enabling us to directly compare performance outcomes of the two training regimes.

#### Participants

Of the 53 naïve participants tested, 3 were excluded from analysis (see *Data analysis* section). Of the remaining 50, mean age was 20 years (range 18–46 years, 11 male). With the exception of one voluntary postgraduate, all were undergraduates who received course credit for their time. None were familiar with the database actors, confirmed during debrief. Prior to this study, approval was obtained from the University’s Research Ethics Committee, and participants provided informed written consent.

#### Stimuli and equipment

Our database comprised 546 ambient facial images of two Italian actors, Luigi Lo Cascio and Fabrizio Gifuni, selected because their prolific film and television careers in Italy provided a wide source of photographic material while neither is well known in the UK.

#### Developing the image database

**Figure 1.**
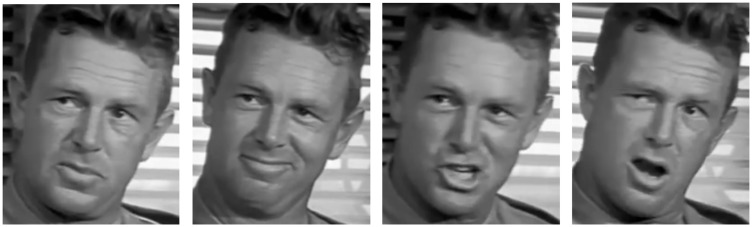
Example of a typical Image Group, featuring the actor Sterling Hayden, taken from the public domain movie ‘Suddenly’ (Bassler & Allen, 1954). Expressiveness ratings, gathered from 40 participants as part of a separate study, are (from left) 42.5%, 58.1%, 54.4% and 66.9%. These images, not used in this study, are for illustrative purposes only.

Images were obtained from screenshots from YouTube clips and the DVDs of 13 movies made between the years 2002 and 2014. As per the method used by Jenkins et al. (2011), images exceeded 150 pixels in height, showed faces free of occlusion, were cropped to portrait dimensions of 4:5 and sized to 320 × 400 pixels. All showed the face from frontal or partial view. Importantly, images were collected in ‘Image Groups’. These are sets of two to nine face images from the same scene, camera and position. This ensured that properties particular to the actors (e.g. facial hair, age) as well as properties specific to the filmed scene (e.g. lighting, camera) were kept largely constant within each set and differed only in expression. Images cannot be reproduced here because of copyright restrictions; however, an illustrative example of a typical Image Group is shown in Figure 1.

To determine image expressiveness, we collected ratings from 40 participants unfamiliar with the faces. They were given the 546 images printed as laminated cards and asked to place each card into one of five boxes labelled from 1 [‘neutral’] to 5 (*very expressive*). The number of the box in which the image was placed was recorded as the score, so images placed in Box 1 were scored as 1, in Box 2 scored as 2, and so forth. The scores from all 40 participants were then summed. This gave us a total score for each image, within the range 40 (i.e. all 40 participants allocating that image to the 1 [‘neutral’] box) to 200 (40 scores of 5 ‘very expressive). For ease of use, we rescaled the range from 40 to 200 to 0 to 100, so that each image expressiveness score was expressed as a percentage, ranging 6.25% to 100% across the database. Participants were instructed to use their judgment and told to put as many or as few images into each box as they wanted. Furthermore, they were given no definition of expressiveness or neutrality; therefore, these terms should be understood in the context of a layperson’s terminology.

#### Stimuli selection

From this database, we selected three image sets: neutral and expressive training sets of 70 images each, and a test set of 208 images (see Table 1).

**Table 1.** Expressiveness of Images per Condition and Number of Times Presented.

	Number of images tested					Expressiveness %		
	Block 1	Block 2	Block 3	Block 4	Total trials	Min.	Max.	Mean (±*SD*)
Phase 1	*70 images seen* × *4 times*							
Neutral training	70	70	70	70	280	9.34	49.34	22.64 (9.18)
Expressive training	70	70	70	70	280	53.75	100	79.22 (11.18)
Phase 2	*208 novel images seen* × *1 time*							
Testing	208	–	–	–	208	6.25	100	42.08 (19.52)

**Figure 2.**
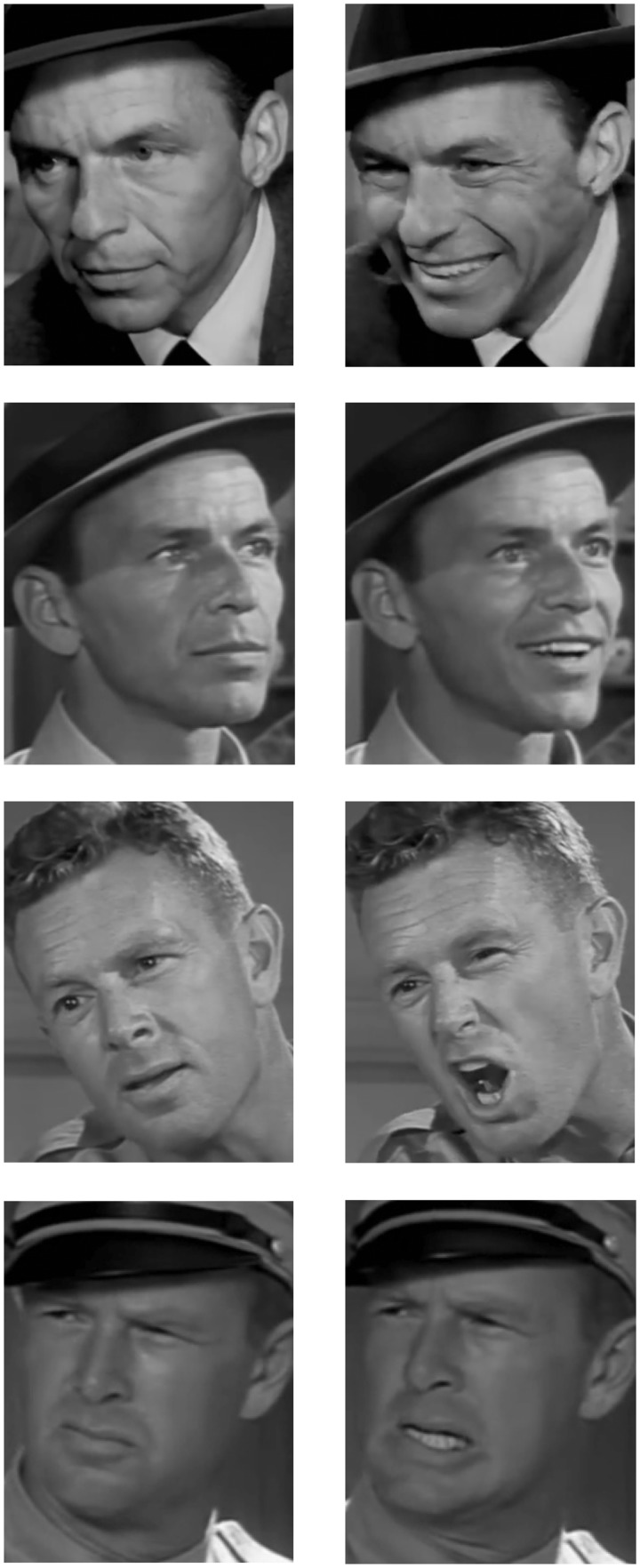
Example illustration of ‘neutral' (left column) and ‘expressive' (right column) counterpart images of actors in the motion picture ‘Suddenly’ (Bassler & Allen, 1954). Top images are of Frank Sinatra; lower images are of Sterling Hayden. Each pair comes from a different Image Group. Expressiveness ratings are (left to right, from top) 17.5%, 77.9%, 14.4%, 77.5%, 29.4%, 89.4%, 48.1% and 70%. These images, not used in this study, are for illustrative purposes only.

To create the training sets, we selected 70 image pairs (35 for each actor), from Image Groups with the highest range of expressiveness. In each pair, one image was low in expressiveness, the other high. We split the 70 pairs into 2 training sets: a neutral set comprising the low-expressiveness images and an expressive set, their high-scoring counterparts. Figure 2 shows illustrative examples.

When selecting the image pairs, we viewed the images themselves, not just their expressiveness scores, so that we could ensure inclusion of all six universal expressions (Ekman & Friesen, 1971) in the expressive training set. We similarly viewed the images during this selection process to ensure as equal a balance as possible between positive (48/70 images) and negative affect expressions (22/70 images), whilst simultaneously selecting pairs that fulfilled the high- and low-scoring counterpart criterion.

The test phase used a further 104 images of each actor, ranging widely in perceived expressiveness. These 208 images were taken from different Image Groups than were the training sets to ensure they did not closely resemble training images.

#### Equipment

Stimuli were presented on a computer monitor with screen resolution of 1280 × 1024 and a refresh rate of 85 Hz. Stimuli were displayed centrally embedded in a 39.3 cd/m^2^ background. There was no fixation point. From the viewing distance of ∼100 cm, stimuli occupied 5.6° × 7.0°. Responses were given on a Microsoft SideWinder gamepad. For training only, feedback was given after each trial; this consisted of either a black tick or cross in the screen centre for 1,000 ms. The experiment was written in MATLAB using the Psychophysics Toolbox extensions (Brainard, 1997; Pelli, 1997).

#### Procedure

The experiment was conducted in a quiet darkened room. For the training phase, the computerised task was to respond quickly and accurately to each face image by making a right key press for ‘Rob’ images and left to indicate ‘Louis’. This phase required responses to 280 trials comprising 4 viewings of 70 different face images, with opportunities for breaks. To avoid the possibility of the same image being presented sequentially, we randomised the pack as follows: For each participant, the set was randomly assigned to Half-set A or Half-set B, each half-set containing 17 images of one actor and 18 of the other. The half-sets were shown in the order, ABABABAB, and image order was randomised within each instantiation of each half-set. This ensured a minimum of 35 images between 2 presentations of an image. After training, participants did a word search followed by the test phase.

#### Data analysis

With the reaction time (RT) data, we analysed the means of the trimmed RT distributions for correct responses. Trimmed means were calculated by taking the untrimmed means and associated standard deviations, and then averaging RTs within two standard deviations of the untrimmed means.

Three participants’ data were excluded from the analyses: one had test phase performance of only 24% suggesting that they had muddled the response keys; and two had proportion-correct z-scores lower than −2 in the training phase final block (proportions correct were 62.3% and 71.0%), indicating some difficulty in learning the faces. Consequently, we recruited an additional three participants, all of whom achieved above-threshold performance.

Initial analysis of mean RT data showed that the homogeneity of variance assumption was compromised; consequently, our analyses are performed on inverse-transformed mean RTs. In our graphs, RTs are transformed back for ease of interpretation.

### Results

#### Training phase

**Figure 3.**
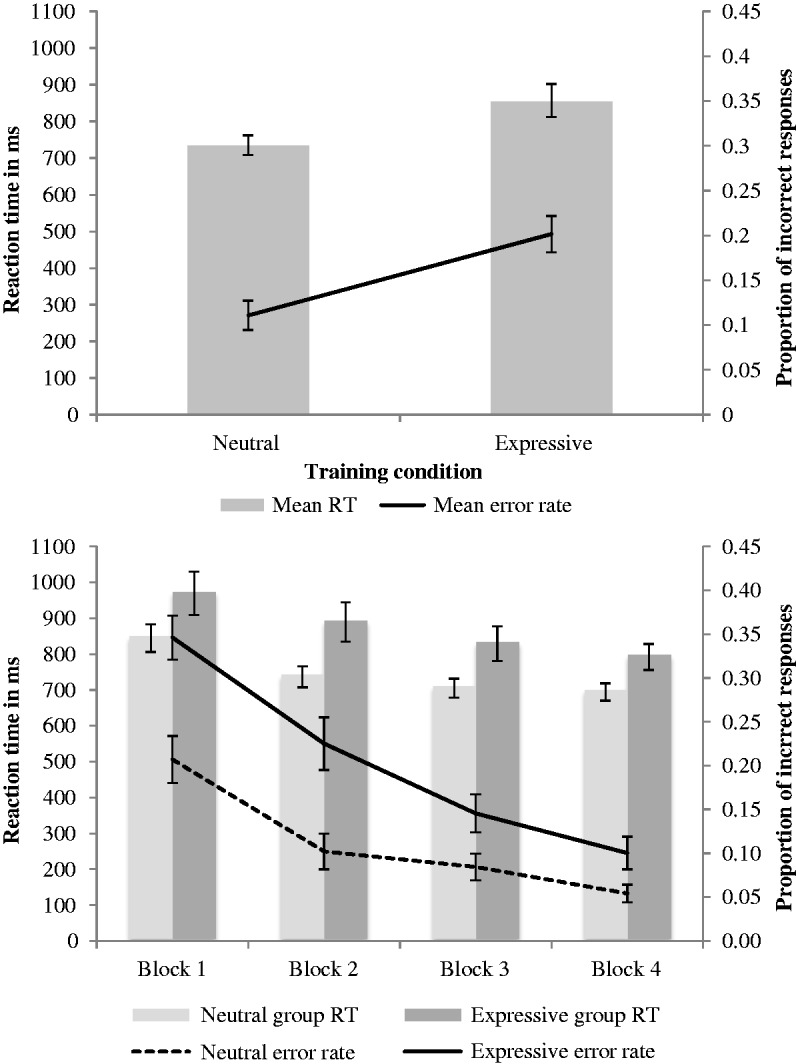
Training phase mean reaction times, plotted with proportion of incorrect responses. *Upper panel:* Data collapsed across training block. *Lower panel:* Data for training blocks in neutral and expressive conditions*.* Error bars denote standard error.

Figure 3 shows the training phase results, overall (upper panel) and by training block (lower panel). Note that in our graphs, we plot both RTs and error rates to demonstrate that RTs are not driven by a speed-accuracy trade-off. Figure 3 (upper panel) suggests that performance was poorer for the expressive training group, and this was supported by the statistical analysis. We conducted a 2 × 4 mixed-design ANOVA of the RT data, with a between-subjects factor of training condition (neutral, expressive) and a within-subjects factor of training block (1, 2, 3, 4). This analysis (Greenhouse-Geisser corrected) revealed the relative superiority of neutral over expressive condition performance, with a significant main effect of training condition, *F*(1, 48) = 5.77, *p* = .020, $\eta_{p}^{2}$ = .107. There was also a significant main effect of training block, *F*(2.28, 109.49) = 49.75, *p* < .001, $\eta_{p}^{2}$ = .509. There was no interaction between training condition and block, *F*(2.28, 109.49) = 1.39, *p* = .250, $\eta_{p}^{2}$ = .028, which is consistent with performance improving under both training regimes.

**Figure 4.**
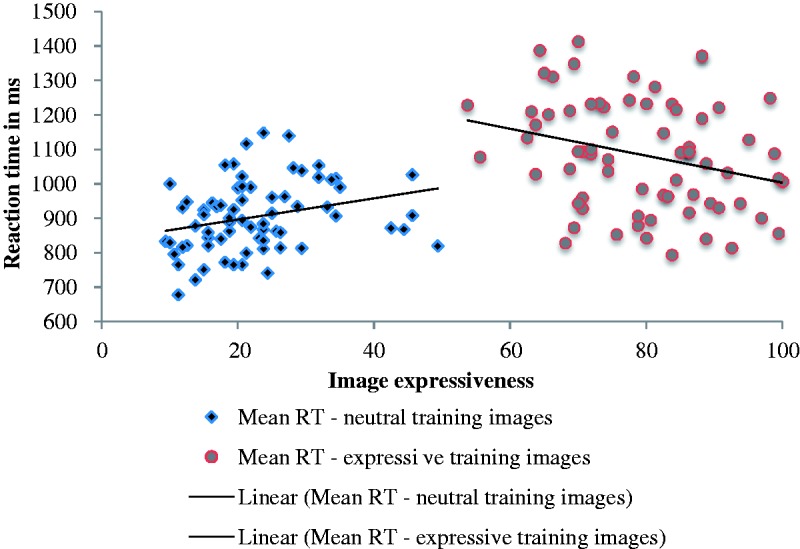
Training phase Block 1: Mean RTs for images in neutral and expressive training, plotted by expressiveness, with lines of best fit.

For both training conditions, we measured the correlation between image expressiveness rating and the mean RT of responses to those images. We did this analysis for the Block 1 responses only, so as to avoid the potential confound of memory for specific images, which may occur because of the repetition of image presentations in Blocks 2 to 4. For the neutral training condition, there was a weak but significant correlation between image expressiveness and mean RTs for those images, *r*(68) = −.29, *p* = .014. Although negative, this correlation is with inverse-transformed data, and therefore indicates that RTs are slower when images are more expressive. This contrasts with the correlation between image expressiveness and the expressive training images, *r*(68) = .27, *p* = .025, which indicates that RTs are faster as image expressiveness increases. Considered together, these correlations suggest that expressiveness has a U-shaped effect on performance; expressiveness correlates with deteriorating performance but at the extreme levels, can be beneficial, perhaps because highly expressive faces can enhance idiosyncrasies, thereby facilitating differentiation. Indeed, this is consistent with Bruce and Young’s (1986, p. 310) suggestion that ‘*characteristic* expressions’ are perhaps important in face recognition. Figure 4 shows the Block 1 mean RTs for images in the neutral and expressive training conditions, plotted by image expressiveness.

#### Test phase

To measure learning, the test phase was the same for all participants irrespective of training condition. Since test images were presented only once, were novel, and came from different Image Groups than the training images, this phase specifically investigated how well identities had been learned.

**Figure 5.**
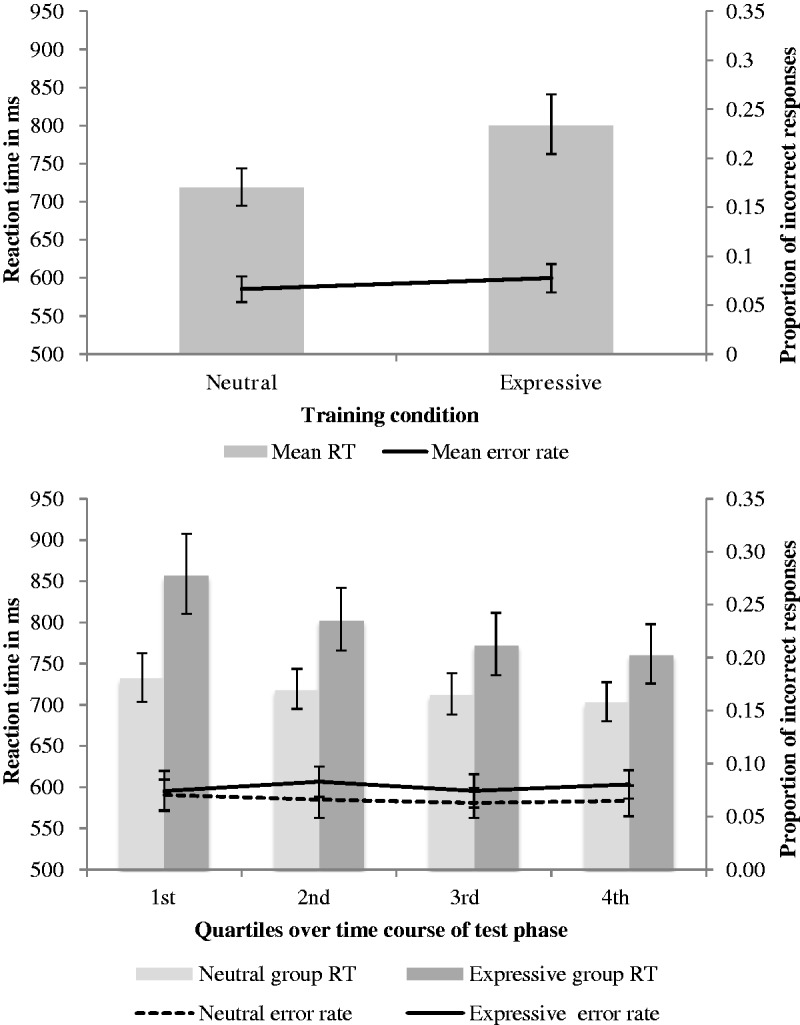
Test phase mean reaction times plotted with proportion incorrect. *Upper panel:* Data for test phase overall. *Lower panel:* Data over time quartiles for neutral and expressive conditions. Error bars denote standard error.

We analysed results by training condition (Figure 5, upper panel) and their time course (Figure 5, lower panel), by means of a 2 × 4 mixed-design ANOVA of the RT data, with a between-subjects factor of training condition (neutral, expressive) and a within-subjects factor of time quartile (1, 2, 3, 4). The analysis (Greenhouse–Geisser corrected) revealed a significant main effect of time quartile, *F*(2.09, 100.20) = 10.89, *p* < .001, $\eta_{p}^{2}$ = .185, demonstrating that participants were continuing to learn during this phase. There was a marginal effect of training condition, *F*(1, 48) = 3.32, *p* = .075, $\eta_{p}^{2}$ = .065, and a borderline interaction between training condition and time quartile where $\eta_{p}^{2}$ indicated a small effect size, *F*(2.09, 100.20) = 2.52, *p* = .084, $\eta_{p}^{2}$ = .050.

For the interaction, follow-up analyses of the simple main effects enabled us to gauge effectiveness of the training regimes for learning by comparing RTs from the first quartile, since performance in these initial trials would be less conflated with the effects of on-going learning. For the first quartile, expressive-trained participants performed significantly worse than the neutral-training group, indicating that expressive training was the inferior regime for face learning, *F*(1, 48) = 5.31, *p* = .026, $\eta_{p}^{2}$ = .100.

To explore performance in relation to image expressiveness, we divided test trials into trials with low-expressiveness images, and those with high. We calculated a paired samples *t*-test comparing RTs to low- and high-expressiveness images, *t*(49) = 1.92, *p* = .061, *d* = 0.33. This clearly suggests that the high-expressiveness images are taking longer to process, but we are unable to draw strong conclusions from a marginal result. Therefore, to investigate whether or not this was the case, we repeated our experiment, but with the neutral condition only since this was the superior training regime. Using the effect size calculated from those data (*d* = 0.33) with alpha level of 0.05 and power of 0.8, we calculated our required sample size of 74+ participants.

## Experiment 2

### Method

#### Participants

Anticipating attrition, we recruited 88 naïve participants and excluded 6 from our analysis (see later). Of the remaining 82 (12 males), mean age was 20 years (range 18–28 years). All were undergraduates who received course credit for their time. All were unfamiliar with the database actors, confirmed during debrief. Prior to this, study approval was obtained from the University’s Research Ethics Committee, and participants provided informed written consent.

#### Procedure and data analysis

We tested in the neutral training condition only, procedure otherwise resembling Experiment 1. We applied the same rejection criteria as for Experiment 1 and excluded the data of one for close-to-chance performance in the test phase (57%). We excluded the data of five for having proportion-correct z-scores lower than −2 in the training phase final block (their proportion-correct scores were 73.5%, 66.2%, 75.8%, 73.9% and 60.0%).

### Results

**Figure 6.**
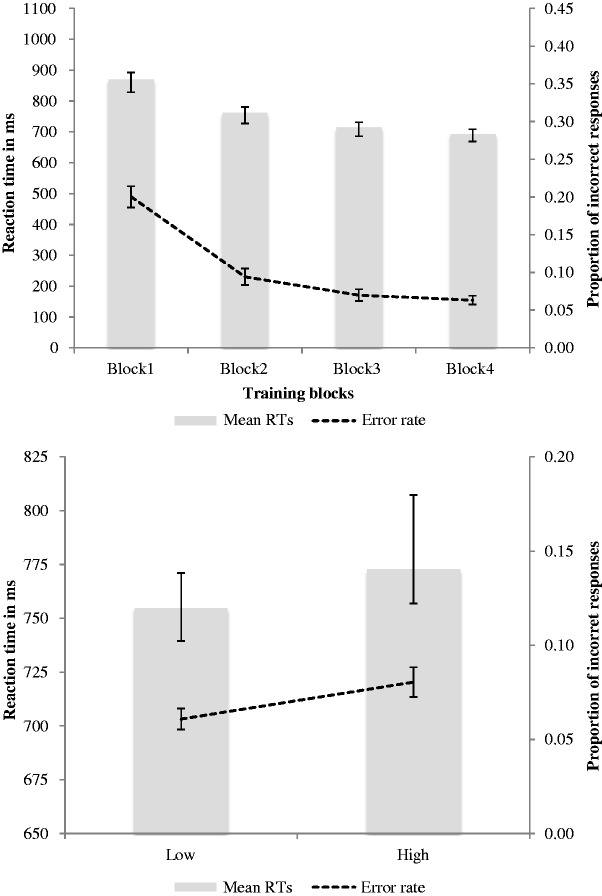
Mean reaction times and accuracy *Upper panel:* Training phase data by block. *Lower panel:* Test phase data by expressiveness of stimuli (low, high). Error bars denote standard error.

Training phase results followed the same pattern as those in Experiment 1, showing improvements in RT and accuracy across blocks (Figure 6, upper panel). Measuring performance in Block 1, there was a weak but significant correlation between image expressiveness and mean RTs to those images, *r*(68) = −.24, *p* = .047. Although negative, this correlation is with inverse-transformed data, and therefore indicates that RTs slowed as image expressiveness increased.

Test phase performance was analysed according to whether stimuli were low or high in expressiveness (Figure 6, lower panel). The mean RT was 18 ms slower in response to high-expressive stimuli compared with low, paired-samples *t*-test *t*(81) = 4.87, *p* < .001, *d* = 0.55. Further, mean proportion correct was 2% lower when stimuli were high compared with low in expressiveness, paired-samples *t*-test, *t*(81) = 3.72, *p* < .001, *d* = 0.46. Both metrics indicate that, consistent with our expectation, performance was inferior when images were of high compared with low-perceived expressiveness.

## General Discussion

Our results show that neutral faces are processed more quickly and with fewer errors than expressive faces, during training (Experiment 1) and at test (Experiment 2). We found, however, some correlational evidence from the first training block of Experiment 1, that recognition task performance for expressive faces improved as they increased in expressiveness. We speculate that this could be attributable to the identities of such faces being more differentiated as expressions become more extreme, due to the exaggeration of idiosyncratic expressions. Performance at the recognition task was dependent on our manipulation of the facial expressiveness of the stimuli.

In our first experiment, we found test performance to be worse after expressive training than after neutral training. One explanation for the poorer test performance of the expressive group is that it was a consequence of not having attained the same level of performance as the neutral group by the end of training. That this occurred as a result of a difference in expressiveness may well be inconsequential; the direct cause of the test difference may well not lie in expressiveness, but simply in the fact that the expressive test material was less well learnt. From the current study, we cannot therefore assume a direct connection between expressiveness of training material and subsequent recognition. However, our experiments do tell us that expressiveness of the training set does modulate training performance, and the expressiveness of the test set does modulate recognition performance.

A convincing explanation for these findings comes from late bifurcation models, which readily predict this task difficulty. They propose that the coding of both changeable and invariant facial aspects occurs in a common framework before visual routes separate for further processing of these characteristics (Calder, 2011). By this account, some aspects of facial identity and expressions are processed by a shared mechanism, which permits the incorporation of changeable facial aspects – such as expressions – into the visual representation of facial identity. This approach is supported by studies reporting such interactions (e.g. de Gelder, Frissen, Barton, & Hadjikhani, 2003; Levy & Bentin, 2008; Van Den Stock & de Gelder, 2014), with evidence suggesting shared coding of identity and expression (e.g. Rhodes et al., 2015), and with findings consistent with expressions being a part of identity representation (e.g. Kaufmann & Schweinberger, 2004). Average-based theories of face representation, in which facial identity representations resemble prototypes that are abstracted from multiple perceptual instances of a face (Burton et al., 2005), are compatible with shared coding and consistent with our findings.

We can propose an alternative explanation of our findings from independent processing models such as Bruce and Young's (1986) and Haxby et al.'s (2000, 2002). These models propose that we recognise identity from processing structural, unchangeable facial aspects separately from the processing of changeable, dynamic aspects such as expressions; and that the bifurcation of facial information into these pathways occurs early. These models might reasonably be extended to incorporate the idea that separating the expressive facial information from the identity-specific structural information is more challenged when faces are expressive. For example, we might suggest that expressions interfere with this process by introducing noise; for example, expressions can alter the appearance of features – such as the shape of the mouth from a smile or the wrinkling of a nose in disgust. By changing the retinal description of the face in this way, expressiveness could slow the extraction of identity-relevant facial information and lead to recognition errors. By this reasoning, these models might explain our findings of expression dependence. However, this interpretation requires some specification of the putative mechanism that separates invariant facial information from changeable aspects.

Another approach to understanding these findings is that they can be explained by image similarity. By this account, training with the expressive faces is slow because these images are less homogenous than the neutral training faces; and when test phase images are expressive, they take longer to respond to because of their dissimilarity to the neutral images with which participants had been trained. Therefore, performance difference is driven by the decreased similarity of expressive images relative to neutral images, and not by image expressiveness per se. Of course, image similarity and expressiveness are intrinsically interrelated. Expressiveness is classified as a changeable aspect of faces (Haxby et al., 2000) and to make an expression we necessarily distort our face; the stronger the expression, the more distorted the face. Consequently, our experiments cannot distinguish whether it is the distortion or the expressiveness itself that is driving the differences observed. What we can say with certainty is that the facial distortion – that we classify as expressiveness – can impede both the learning and recognition of facial identity.

In conclusion, this study demonstrates that facial expressiveness modulates identity processing: it hinders identity discriminations of unfamiliar and newly learned faces and impedes facial identity learning; and when the faces being learned are highly expressive, then expressiveness may improve rather than impede performance, possibly by enhancement of distinguishing facial features. By demonstrating expression dependence, our results fit with the view that facial expressions are not disregarded or parsed out by our identity-learning mechanism, but interact with it. Our findings contribute to the body of evidence that refutes independent processing of identity and expressions. Moreover, these results lend support to Calder's (2011) late bifurcation model of face processing in which changeable facial aspects and identity are coded in a common framework and, consequently, are consistent with the concept that expressions form an integral part of facial identity representation.
